# Towards international collaboration of clinical research networks for EMDR: the EMDR Pain Network Germany

**DOI:** 10.3389/fpsyg.2024.1449150

**Published:** 2024-10-04

**Authors:** S. Vock, A. Hofmann, M. Lehnung, K. Böhm, S. Wieland, G. H. Seidler, E. Beiner, M. Hermes, H.-C. Friederich, W. Eich, J. Tesarz

**Affiliations:** ^1^Department of General Internal Medicine and Psychosomatics, Heidelberg University, Heidelberg, Germany; ^2^EMDR-Institute, Eckernfoerde, Germany; ^3^Private Practitioner, Eckernfoerde, Germany; ^4^EMDRIA Germany e.V., Freiburg, Germany; ^5^Psychotraumatologie Praxis Heidelberg, Heidelberg, Germany; ^6^DZPG (German Centre for Mental Health – Partner Site Heidelberg/Mannheim/Ulm), Berlin, Germany; ^7^Department of Psychosomatic Medicine and Psychotherapy, University Medical Center of the Johannes Gutenberg University Mainz, Mainz, Germany

**Keywords:** chronic pain, eye movement desensitization reprocessing, EMDR, therapy, network

## Abstract

**Background:**

Eye Movement Desensitization and Reprocessing (EMDR) is an evidence-based treatment, primarily established for post-traumatic stress disorder (PTSD). While it is increasingly being applied to chronic pain, its efficacy in this area is not yet supported by the same level of evidence as that which exists for PTSD. Studies in this area often show heterogeneous results with small case numbers, and the potential side effects of EMDR in the treatment of chronic pain are not well understood. Systematic documentation of treatment effects, potential predictors of treatment response and non-response, and side effects is crucial for progress in this field.

**Aim:**

The primary aim is to establish a research framework to systematically investigate the delivery of EMDR therapies by outpatient clinicians in the field of pain. This study aims to provide a comprehensive analysis of treatment outcomes, side effects and determinants of treatment effectiveness, whether positive response or non-response.

**Methods:**

This framework will oversee the documentation and evaluation of EMDR interventions delivered in outpatient settings using an Embedded Continuous Cumulative Evaluation Design (ECCED). It will focus on detailed characterisation of positive and negative therapeutic effects. It will also identify and analyse prognostic factors that influence individual variability in response to treatment. Treatment materials, standardised assessments and an intervision platform for regular exchange will be provided.

**Discussion:**

The establishment of the EMDR Pain Network Germany and an interdisciplinary scientific-clinical platform is essential to promote clinical exchange and understanding of the effects of EMDR in pain therapy. This platform offers standardised treatment protocols, an online data collection system with anonymised data, comprehensive baseline assessments and an intervision platform for regular exchange. The knowledge gained is intended to personalise future therapies and serve as a basis for large randomised clinical trials.

## 1 Introduction

Worldwide, pain represents a significant health problem affecting millions of people. Demographic changes and the increasing prevalence of chronic diseases have led to a rise in pain cases, resulting in a growing societal burden (Vos et al., 2020; Wettstein and Tesarz, 2023). It is estimated that more than 20% of adults worldwide suffer from chronic pain, and the number of new people suffering from this condition is increasing every year. This makes chronic pain one of the leading causes of long-term disability worldwide (Gatchel et al., 2007). Despite various effective therapeutic approaches for treating acute pain, therapy for chronic pain often remains insufficient, leading to significant impairments and reduced quality of life for those affected (Vos et al., 2020). The persistence of these pain conditions highlights the limitations of current treatment methods and the need for innovative approaches that address pain as a complex biopsychosocial phenomenon (Pinto et al., 2023).

A promising approach in the treatment of chronic pain is Eye Movement Desensitization and Reprocessing (EMDR), a method originally developed for treating post-traumatic stress disorder (PTSD). In recent years, the scope of EMDR has expanded beyond PTSD therapy, particularly in the treatment of chronic pain syndromes. This expansion is due to the potential of the method to target not only the pain symptoms themselves but also the associated psychological and emotional components such as anxiety, depression, and avoidance behaviour (Gilam et al., 2020; Hashmi et al., 2013; Matthijssen et al., 2020; Pinto et al., 2023).

Several randomised controlled trials have demonstrated the effectiveness of EMDR in various chronic pain syndromes, including musculoskeletal pain (Gerhardt et al., 2016), back pain (Gerhardt et al., 2016), headaches (Konuk et al., 2011), phantom pain (Rostaminejad et al., 2017), fibromyalgia (Borst et al., 2024; Friedberg, 2004; Zat Çiftçi et al., 2024), and rheumatoid arthritis (Matthijssen et al., 2020; Tesarz et al., 2019). The results of these studies indicate that EMDR can significantly reduce pain intensity and lead to a decrease in pain-related impairments and psychological distress (Leisner et al., 2014).

A key aspect that distinguishes EMDR from traditional pain therapeutic approaches is its direct analgesic effect. While conventional approaches often achieve only indirect effects by reducing anxiety and depression, more recent studies suggest that EMDR significantly reduces both pain symptoms and the extent of pain-related impairments. Additionally, the results indicate partially stable long-term effects of up to 2 years after the completion of therapy (Tesarz et al., 2014). These positive effects are attributed to the modulation of neural processes involved in both emotional and pain-related processing.

Research shows that in chronic pain, an “emotional shift” often occurs, where pain is no longer processed in the classical pain-processing regions of the brain but rather in the emotion-processing areas (Hashmi et al., 2013).This mechanism has parallels to the “flashbacks” experienced by trauma patients, making the use of EMDR in chronic pain particularly plausible.

At the same time, studies in this field also show heterogeneous results, often due to small sample sizes and a limited understanding of potential side effects (Matthijssen et al., 2020; Tesarz et al., 2013). These challenges underscore the need for a systematic approach to documenting and analysing treatment outcomes. Establishing regional networks to promote information exchange and systematically analyse the implementation of EMDR therapies in practice could significantly contribute to optimizing the use of EMDR in chronic pain.

The complexity of psychotherapeutic interventions and their outcomes in clinical studies also presents a significant challenge, particularly in terms of the reproducibility and generalisability of results (Lutz, 2003). Conventional randomized controlled trials often do not fully capture the nuances of psychotherapy, leading to a gap between research findings and clinical practice. Close collaboration between researchers and therapists could bridge this gap and ensure that research findings are practical and patient-centred (Asay et al., 2002).Given the time and cost constraints of psychotherapy research, which often lead to studies with small sample sizes (Williams et al., 2012), a collaborative research network promotes a continuous dialogue between researchers and therapists. This could help integrate new insights into clinical practice more quickly and improve the treatment of chronic pain through EMDR.

### 1.1 Objectives

The ‘EMDR Pain Network Germany’ was founded with the aim of promoting and further developing the quality and scientific evidence of EMDR in pain therapy in accordance with the current state of research and to support the exchange between clinicians and thus promote and scientifically accompany the implementation of EMDR in the therapy of chronic pain. This network is a scientific cooperation project under the direction of the Department of General Internal Medicine and Psychosomatics at Heidelberg University Hospital in collaboration with the professional association EMDRIA Deutschland e.V. and the EMDR Institute Germany. It aims to facilitate (1) longitudinal research into the therapeutic effects of EMDR in the treatment of chronic pain at an individual level, including adverse effects, (2) the identification of potential predictors of individual response/non-response to treatment between participants, and (3) the facilitation and scientific monitoring of the implementation of EMDR in pain therapy.

## 2 Design and procedure

This research initiative will thoroughly assess the implementation of Eye Movement Desensitization and Reprocessing (EMDR) therapies by outpatient clinicians. We will conduct a detailed follow-up that evaluates treatment results, documents adverse events, and identifies key factors influencing treatment effectiveness, which encompasses both positive responses and non-responses. Furthermore, we will promote the broad implementation and practical use of EMDR in managing pain. The ‘EMDR Pain Network Germany’ provides therapists with the resources they need to deliver effective EMDR treatments. Our standardised protocols, training materials and platform for professional interaction ensure uniformity and excellence in therapeutic practices, improving the effectiveness of EMDR treatments across diverse clinical environments.

In accordance with the ethical principles governing human research, all participants and therapists are required to provide written informed consent prior to enrolment. Ethical approval for data collection was obtained from the Research Ethics Committee II of the Medical Faculty of the University of Heidelberg (approval number S-696/2023), ensuring compliance with the ethical standards of the Declaration of Helsinki.

### 2.1 Material

This study is an accompanying evaluation conducted in conjunction with the establishment and expansion of a national network for EMDR therapy focused on chronic pain management. Utilizing an Embedded Continuous Cumulative Evaluation Design (ECCED), the core scientific objective of this network is to carry out a comprehensive, real-time assessment of EMDR therapy as delivered by independent therapists in private healthcare settings. The ECCED represents an innovative research framework designed to seamlessly integrate evaluation into routine clinical practice. Developed as part of the PerPAIN research network (Beiner et al., 2022), it draws inspiration from existing cumulative assessment models, particularly those used in educational research (Kerdijk et al., 2013). This approach allows for ongoing and cumulative data collection throughout the therapeutic process, without a predetermined endpoint. The ECCED is crafted to ensure minimal disruption to the therapeutic process while facilitating continuous monitoring of both therapeutic outcomes and any adverse events that may occur.

In practice, ECCED embeds evaluation mechanisms directly into the everyday clinical activities, ensuring that data collection is a natural part of the therapy rather than an external add-on. Data is collected continuously, allowing for real-time tracking of patient progress and the identification of trends and patterns over time. This cumulative data collection provides a thorough understanding of treatment efficacy and safety. The design of ECCED emphasizes standardized procedures to maintain consistency and comparability across different settings and patients. Detailed descriptive analysis of therapeutic outcomes and adverse events is a key component, alongside systematic research into prognostic factors that may influence treatment responses. This approach enables the differentiation between positive responses and non-responses to therapy.

To guide future research and enhance comparability, a core assessment framework is proposed (see Table 1). This framework aims to standardize evaluations and facilitate data pooling, ultimately contributing to improved research quality and the optimization of EMDR therapy for chronic pain.

**Table 1:** Measures applied at different points of data acquisition.

Instruments	Description (number of items, time for completion)	Time for completion		
		T_0_	T_1_	T_2_–T_4_
Baseline characterization				
Basis sociodemographic data (Bado)	Assessment of basic sociodemographic data on age, gender, nationality (German/ other), marital status (living with a partner; yes/ no), educational level (International Standard Classification of Education, ISCED ≤2 and professional life (paid employment/ disability pension/ old-age pension) (Heuft et al., 1998)	X		
Pre-treatment assessment	Standardized assessment of previous and current analgesic treatments	X		
Patients’ Therapy Expectations and Evaluations (PATHEV)	Questionnaire on therapy expectation and evaluation (11 items) (Schulte, 2005)	X		
Baseline assessment of overall level of stress and illness experience, Evaluation of psychosomatic symptoms				
Somatic Symptom Disorder (SSD-12)	12-item questionnaire to assess patient’s perceptions of symptom-related thought, feelings and behaviours (Toussaint et al., 2016)	X	X	
Symptom Severity Scale (SSS-8)	8-item inventory to assess the burden of somatic symptoms (Gierk et al., 2014)	X		
Short-Form-Health Survey 12 (SF-12)	Measure of impact of physical and mental health status on everyday life (12 items) (Gandhi et al., 2001)	X	X	X
Perceived Stress Scale (PSS)	Assessment of perceived stress (10 items) (Klein et al., 2016)	X	X	
Childhood Trauma Questionnaire (CTQ)	Questionnaire to assess early adversities specifying 5 dimensions (emotional, physical and sexual abuse, physical and emotional neglect) (28 items) (Bernstein et al., 1998)	X		
Resilience Scale (RS11)	11-item questionnaire to identify the degree of individual resistance (Kocalevent et al., 2015)	X	X	
Pittsburgh Sleep Quality Index (PSQI)	Questionnaire to assess sleep quality (19 items) (Buysse et al., 1991)	X	X	
Psychological comorbidities				
Hospital Anxiety and Depression Scale (HADS)	Assessment of the level of anxiety and depression (14 items) (Zigmond and Snaith, 1983)	X	X	X
Positive and Negative Affect Schedule (PANAS)	20-item assessment to measure positive and negative affects (Watson et al., 1988)	X	X	
Somatoform disorders, unclear physical complaints				
Whiteley Index-7 (WI-7)	Measure of the level of illness anxiety to assess somatization and hypochondriasis (Glöckner-Rist et al., 2007)	X	X	
Fatigue Severity Scale (FSS)	Questionnaire to assess the impact of fatigue (9 items) (Krupp et al., 1989)	X	X	
Chronic pain				
Fear-Avoidance Beliefs Questionnaire (FABQ)	Questionnaire to assess fair of pain and avoidance of physical activity (16 items) (Pfingsten et al., 2000)	X	X	
Fear of Pain Questionnaire (FPQ)	Measure to assess fear of different stimuli usually causing pain (30 items) (McNeil and Rainwater, 1998)	X	X	
Widespread Pain Index (WPI)	Assessment of the spatial extent of pain (distinguishing between 19 body areas and 5 body regions) (Galvez-Sánchez et al., 2020)	X	X	
Multidimensional Pain Inventory (MPI-D)	12-scale inventory to assess important dimensions of chronic pain experience (52 items) (Kerns et al., 1985)	X	X	X
Chronic Pain Grade Scale (CPG)	7-item measure designed to evaluate pain intensity and pain-related disability in adults with chronic pain conditions (7 items) (Dixon et al., 2007)	X	X	
Pain-Related Self Statement Scale (PRSS)	Measure to assess situation-specific aspects of patients’ cognitive coping with pain (18 items) (Flor et al., 1993)	X	X	
Western Ontario and McMaster Universities Arthritis Index (WOMAC)	Assessment of pain, stiffness and functional impairment (24 items) (Bellamy, 2005)	X	X	
Trier Inventory of Chronic Pain (TICS)	The questionnaire measures six aspects of chronic stress (57 items) (Schulz et al., 2004)	X		
Brief Symptom Inventory (BSI)	Questionnaire to record subjective impairment due to physical and psychological symptoms (53 items) (Geisheim et al., 2002)	X		
Pain Sensation Scale (Schmerzempfindungsskala, SES)	24-item questionnaire to measure and differentiate description of subjectively perceived pain (Geissner, 1995)	X	X	
Past stress experiences and post-traumatic distress				
Critical life events and life crises (Kritische Lebensereignisse und Lebenskrisen, KLL)	32-item inventory to assess critical life events and life crises (Tennant and Andrews, 1976)	X		
Posttraumatic Diagnostic Scale (PDS)	Measure to assess PTSD symptom severity in the last month (24-items) (Foa et al., 1997)	X		
Somatic illnesses and high psychological stress				
Oswestry-Disability Inventory (ODI)	Questionnaire to assess pain-related functional restrictions in patients with back pain (10 items) (Fairbank and Pynsent, 2000)	X	X	X
Compliance Questionnaire for Rheumatology (CQR5)	5-item questionnaire measuring compliance with rheumatic disease treatment (Hughes et al., 2013)	X	X	
Study-specific evaluation form				
Negative Effects Questionnaire (NEQ)	Questionnaire to assess unwanted and negative effects of the treatment (32 items) (Rozental et al., 2016)		X	
Inventory for Assessing Negative Effects of Psychotherapy (INEP)	Questionnaire to assess unwanted and negative effects of the treatment (21 Items) (Ladwig et al., 2014)		X	
Patient’s global impression of change (PGIC)	Patient’s global impression of change (1-items) (Hurst and Bolton, 2004)		X	X
Additional Patient-Reported Outcome Measures (PROMs)	Assessment of Change in medication after treatment (1-item) (Weldring and Smith, 2013)		X	X
Agnew Relationship Measure Patient (ARM-5)	5-item questionnaire about client-therapist alliance (Cahill et al., 2012)		X	

### 2.2 Pain focused EMDR therapy

EMDR therapy is a stress-reducing intervention that combines proven trauma intervention elements such as imaginal exposure, cognitive and self-control techniques with specific techniques such as bilateral sensory stimulation (e.g., left–right eye movements or bilateral tapping with the therapist’s hand). This is done according to the principle of dual focus of attention (Schubert et al., 2011). This principle describes how patients simultaneously focus on distressing memories and an external bilateral sensory stimulus (Laliotis et al., 2021). This process appears to facilitate the processing of emotionally distressing memories (e.g., traumatic events or pain sensations) and reduce or even eliminate the emotional distress associated with these memories (Laliotis et al., 2021). During each EMDR session, the patient deals with distressing traumatic or pain-related memories and the associated feelings, cognitions and body sensations while focusing on a series of external bilateral stimuli. In the subsequent phase, the patient’s attention is directed towards the emergence of new associations, which are then subjected to further scrutiny through a series of dual attention exercises. This process of dual attention and personal association is repeated throughout the session until the original target (trauma or pain-related memories) is no longer a significant source of distraction. The duration of a single session typically ranges from 50 to 90 min. All additional treatments are recorded in detail (Table 2, for more details see also Supplementary material). The therapists are afforded considerable flexibility in the organisation of their EMDR sessions, with the respective contents and potential deviations being briefly documented. However, in order to standardise case conceptualisation and treatment implementation, all participating therapists receive a treatment protocol and manual for orientation. The treatment protocol for this study is based on a standardised manual (Tesarz et al., 2015), and the possible goals for treatment include disturbing memories, current pain perceptions and pain-related fears and cognitions.

**Table 2:** Therapists’ documentation.

Measures for therapists		Time for completion
Standardized EMDR treatment documentation	Standard case report forms for therapists (e.g., change in subjective level of distress during session, treatment targets, adverse events, etc.)	For each session
Severe adverse events (SAE)	Assessment of severe adverse events and their relationship to therapy (6 items)	For each session
Therapist questionnaire	6-item questionnaire about therapists’ experience with EMDR	T_0_
Therapists’ global impression of change (TGIC)	2-item questionnaire about therapists’ global impression of change of their patients (Ferguson and Scheman, 2009)	T_1_
Agnew relationship measure therapist (ARM-5)	Questionnaire about client-therapist alliance (5 items) (Cahill et al., 2012)	T_1_

The proposed case conceptualisation in the treatment of chronic pain follows a three-stage approach according to the standard model of past, present and future, distinguishing between pain-related and non-pain-related trauma.

The classic protocol provides for a systematised target selection, in which, in cases in where the pain is associated with traumatic and (even today!) stressful experiences, desensitisation and reprocessing of these experiences should be started first (Borst et al., 2024; Tesarz et al., 2015). In cases where either no traumatic experience can be identified as the trigger or where the traumatic event has been reprocessed more successfully in the meantime, the distressing memories and thoughts associated with the pain should be processed. After processing distressing memories and thoughts, and in cases where no distressing memories or thoughts can be identified initially, the pain can also be focussed on directly using the specific pain protocol. In cases where patients gain access to new, previously repressed experiences during pain processing, reactivated memories can be revisited and further processed during the course of therapy using the standard protocol or a modified standard protocol. Finally, or in addition, it is possible to use the standard protocol, modified standard protocol, the absorption exercise or the flash-forward protocol to alleviate dysfunctional fears of illness, future pain crises and potential pain triggers.

In practice, however, it has been shown that other targets are relevant in the treatment of patients with pain in addition to classic traumas and the pain itself.

For example, interpersonal conflicts and maladaptive health fears often play also a central role in patients with chronic pain. Thus, in contrast to patients with post-traumatic stress symptoms, the focus of treatment for patients with chronic pain is often initially on the present. This includes processing the current pain as well as dealing with pain-related fears, with a particular focus on dysfunctional health fears. Health fears reinforce the perception of the threat posed by one’s own body, whereby it is not only about the fear of possible illnesses, but also about frightening ‘misconceptions’ about one’s own body (e.g., ‘damaged intervertebral disc’ or ‘worn joint surface’). It is of the utmost importance to ascertain the patient’s individual understanding of the illness, to identify defect-oriented perceptions of their own body and, if necessary, to correct them so that the patient can regain confidence in their own body. Furthermore, relevant interpersonal conflict situations of the patient are considered in treatment planning. The network deliberately avoids standardised guidelines for case conceptualisation in order to reflect the real-world setting as well as possible and at the same time not to restrict the therapists too much. Instead, systematic documentation of the target selection by the therapist is planned in order to be able to explore possible peculiarities in the case conceptualisation in greater depth.

### 2.3 Procedure

**Figure 1:**
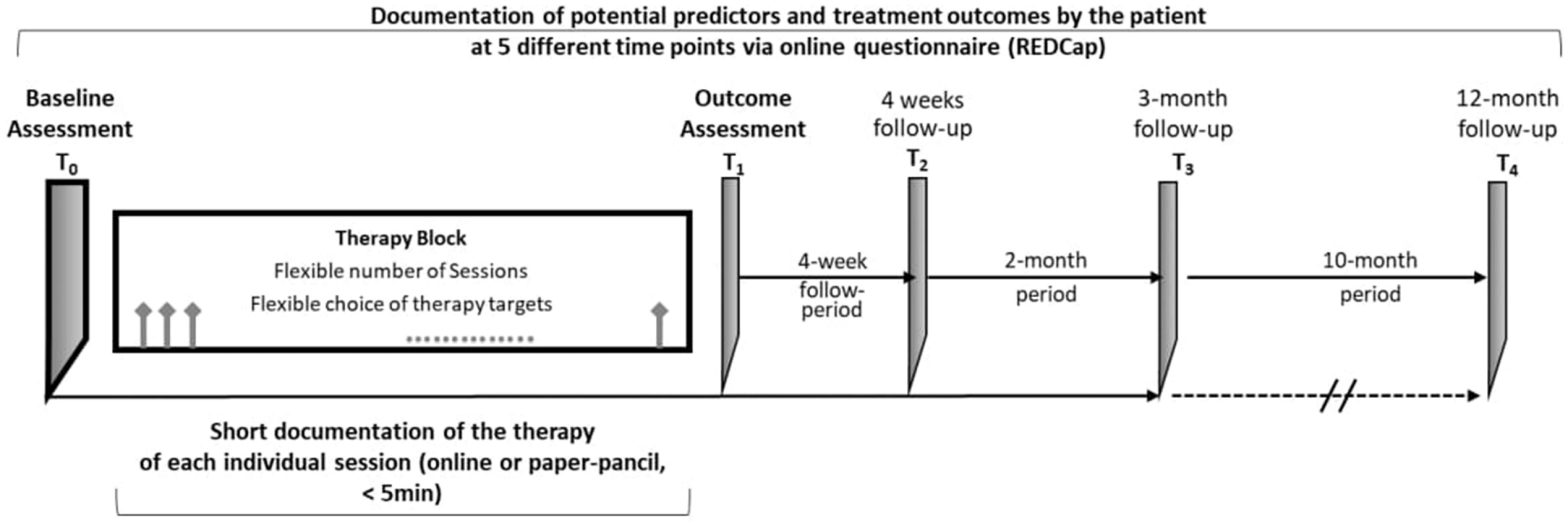
Flow chart of data acquisition. T_0_, baseline assessment; T_1_, post-treatment outcome assessment; T_2_–T_4_, 4-month follow-up assessment.

Outpatient therapists will identify patients with chronic pain conditions for whom they would like to provide an EMDR intervention as part of their regular psychotherapy. In a first step, these patients will be informed by the therapists and will give their written consent to be contacted by the study team at the University Hospital of Heidelberg. There will be no specific recruitment; rather, therapists will decide which patients to admit for outpatient therapy and which to report to the EMDR network. Within a further diagnostic appointment, the Structured Clinical Interview for the Diagnostic and Statistical Manual for Mental Disorders (SCID-5) (First et al., 2015) will be conducted by trained research assistants via telephone. Figure 1 illustrates the subsequent steps of data acquisition, which will be conducted online using the web application REDCap (Harris et al., 2019). First, eligible patients undergo a comprehensive baseline assessment (T_0_), including a broad range of self-rating instruments on various domains of relevant symptomatology, physical and emotional functioning, and other potential mechanistic and predictive factors. When patients and therapists decide to end the therapy, we will conduct a post-treatment outcome assessment (T_1_) including main outcomes, relevant measures from the baseline assessment, therapeutic alliance, therapy evaluation, and potential adverse effects associated with the treatment. Finally, three follow-up assessments (T_2_–T_4_) will be implemented 4 weeks and 3 months and 12 months post-treatment.

During the treatment period, therapists will meticulously document each therapy session to ensure comprehensive data collection and analysis. This documentation includes recording severe potential adverse events (e.g., hospital admissions, suicidality) and specific features of the EMDR treatment, such as the therapeutic target, the bilateral stimulation technique used, and the subjective units of distress. Additionally, therapists will complete a questionnaire regarding their prior experience with EMDR and the frequency with which they treat patients with chronic pain.

The documentation process involves capturing detailed information on critical aspects of each EMDR session, including the patient’s distress levels and beliefs about their symptoms before and after the session. Therapists will also assess and record the patient’s acceptance of the psychological model and provide a brief summary of the session’s content.

By systematically documenting these elements, therapists will create a comprehensive data set that facilitates detailed analysis of the processes and outcomes of EMDR therapy. This rigorous approach enhances the validity of the trial and contributes to the growing evidence base for EMDR therapy in the treatment of chronic pain and associated psychological conditions.

### 2.4 Sample

The network includes both patients and therapists, each meeting specific inclusion and exclusion criteria to ensure the reliability and validity of the study outcomes.

#### 2.4.1 Inclusion criteria

- Therapists: Must have completed formal training in psychotherapy and possess an officially recognized certification in EMDR therapy. Only therapists who meet these stringent professional qualifications are included to ensure the consistency and quality of EMDR delivery.
- Patients: Eligible patients are those who have sufficient proficiency in the German language, as effective communication is essential for the therapeutic process and for providing informed consent. Patients must report experiencing chronic pain and must be commencing therapy with a therapist participating in this network. The prerequisite is that the patients have already started psychotherapy, but the planned EMDR treatment has not yet taken place as part of this therapy but is planned in the further course of therapy. Completed previous therapies, including EMDR, are not an exclusion criterion, but will be documented systematically. Patients must be at least 18 years old, with no upper age limit, and be able to give informed consent.

#### 2.4.2 Exclusion criteria for patients

- Severe Psychiatric Comorbidity: Patients exhibiting severe psychiatric conditions that impair their ability to provide informed consent, including suicidality and psychosis spectrum disorders, are excluded. These conditions can significantly interfere with the patient’s ability to participate effectively in the study and can complicate the assessment of EMDR’s efficacy.
- Language Proficiency: Patients lacking sufficient proficiency in the German language are excluded to ensure clear communication and accurate understanding of the therapy and study requirements. This criterion is critical for maintaining the integrity of the therapeutic process and the reliability of the study data.

By establishing these inclusion and exclusion criteria, the study aims to create a representative sample of patients with chronic pain that can provide robust and reliable data on the efficacy of EMDR therapy in treating chronic pain. The dual focus on therapists’ qualifications and patients’ characteristics ensures a high standard of therapeutic intervention and data collection. This methodological rigour is essential for drawing valid conclusions about the therapeutic benefits of EMDR in this specific patient population.

### 2.5 Sample size calculation

The sample size calculation for the present exploratory and feasibility evaluation within the ‘EMDR Pain Therapy Network’ is primarily based on the need to obtain meaningful data on the feasibility and efficiency of the network structures. Considering the aim of assessing the feasibility of a possible future randomised controlled trial, a minimum number of 20 therapists will be recruited, each of whom should enrol at least 10 patients in the trial. This results in a total of at least 200 patients.

This sample size is appropriate for the following reasons:

- It enables a comprehensive assessment of the efficacy and side effect profiles of EMDR therapy for chronic pain.
- It is suitable for testing the feasibility of an accompanying evaluation in the outpatient setting, including documentation quality and patient satisfaction.

The results of the evaluation of the initial sample of 200 patients will be crucial in planning a larger multicentre trial. They should show whether the network is able to recruit enough patients and provide high quality data, and whether the therapeutic practices within the network are sufficiently standardised to conduct a randomised controlled trial.

It is important to note that this number of cases does not have to be the definitive end of the trial. Rather, once this initial target number has been reached, an interim analysis should be carried out to assess the logistical and administrative aspects of the study implementation, in addition to the scientific aspects. On this basis, an adapted or expanded recruitment strategy can be developed for the next phase of the study.

## 3 Advancing implementation of EMDR in clinical practice

Therapists participating in the study will be recruited via the EMDRIA Germany or will be referred by other participating therapists. Additionally, the project will presented at national and international conferences, gaining exposure to a broader audience, and an article about the project has been published in the EMDRIA journal. The study is open to all psychotherapists and physicians with formal psychotherapeutic training and accredited EMDR certification.

To promote the implementation of EMDR in pain therapy, the network emphasises supporting the application of EMDR in pain management according to scientific standards. This is achieved by providing validated EMDR treatment protocols free of charge (see Supplementary material) and offering practical working materials for therapists. These measures not only enhance the appeal of network membership but also encourage the standardisation of treatment approaches and facilitate the specialisation of EMDR therapists in treating patients with complex pain disorders.

Additionally, the network has launched a virtual platform where therapists can connect, share experiences, and participate in bimonthly online peer supervision meetings. During these meetings, they discuss complex patient cases and receive supervisory feedback. The platform is supplemented by a regularly updated newsletter that informs about the latest developments, research findings, and relevant events in the field of pain therapy. The newsletter provides lay-friendly summaries of current scientific studies and contextualises them within the clinical setting, thus aiding the translation of scientific knowledge into clinical practice.

## 4 Outcomes

### 4.1 Patients variables

#### 4.1.1 Treatment outcomes and efficacy

In this study, we aim to identify factors that predict a significant treatment response to EMDR therapy for chronic pain. We will follow established recommendations for outcome measures in clinical chronic pain trials and recommendations for interpreting clinical importance of group differences [Initiative on Methods, Measurement, and Pain Assessment in Clinical Trials, IMMPACT (Dworkin et al., 2008; Turk et al., 2003)]. Core outcome domains include pain intensity, physical functioning, emotional functioning, stress experience, and both patient and therapist ratings of patients improvement and satisfaction.

The effectiveness of EMDR therapy will be quantified by determining effect sizes and response and non-response rates on various outcome variables.

#### 4.1.2 Patient perception of change

Global ratings of change of the overall situation due to treatment was evaluated using the Patient Global Impression of Change (PGIC) scale, which is a single-item rating on a 7-point scale ranging from “very much improved” to “very much worse” (Ferguson and Scheman, 2009). Changes of “much improved” and “very much improved” are considered as clinically relevant. Similarly, therapists should perceive that at patients show at least 60% of improvement, as measured by a score of greater than or equal to 5 on the Therapist Global Impression of Change Scale (TGIC).

#### 4.1.3 Predictors of treatment outcome

A multidimensional phenotyping of patients will be performed, including various aspects such as clinical symptoms, psychological comorbidity, personality factors, therapy expectations and functionality. The aim is to identify predictors that can be used to predict treatment success or failure.

#### 4.1.4 Evaluation of feasibility and practicability

The EMDR Pain Network will evaluate the structures and processes of the network for conducting larger multi-centre clinical trials. This will include the assessment of inclusion rates per therapist, the quality of documentation of outcome variables, and patient satisfaction with the adjunctive evaluation.

#### 4.1.5 Adverse effects associated to EMDR therapy and safety outcomes

1. Negative Effects: These are intended to be captured using the Negative Effects Questionnaire (NEQ) and the Inventory for Assessing Negative Effects of Psychotherapy (INEP).

2. Severe Adverse Events (SAEs): These will be documented and examined for potential causal relationships with EMDR therapy.

#### 4.1.6 Comprehensive assessment

Table 1 shows all measures used during the different points of data acquisition. In addition to the assessment of core-outcome domains, an extensive baseline assessment is performed using standardised and validated questionnaires (see Table 1). The aim is to identify possible predictors in the areas of psychological comorbidity (anxiety, depression, somatization and sleep disorders), catastrophizing, fear of illness and fear avoidance behaviour, past stress experiences and post-traumatic distress, therapy expectations and therapy experience, as well as individual coping and resilience factors.

These defined criteria should not only capture the direct benefits and tolerability of EMDR therapy in chronic pain patients, but also contribute to the optimization of therapeutic approaches and the improvement of treatment quality.

### 4.2 Statistical analyses overview

The primary aim of the ‘EMDR Pain Therapy Network’ is to generate a high-quality dataset that facilitates the investigation of future effectiveness measures, response rates, and potential predictors for both response and non-response. This dataset will provide broad opportunities for exploratory analyses to address future research questions. Within the network, specific questions and analyses are pre-planned:

Descriptive Analysis: To characterise the sample, descriptive statistics will be employed, covering demographic data, clinical characteristics, and outcome criteria. Continuous variables will be described using means, standard deviations, medians, and interquartile ranges. Categorical variables will be presented through frequencies and percentages.
Effectiveness Outcome Analysis: The effectiveness of EMDR therapy will be evaluated by calculating effect sizes using Cohen’s d, with t-tests to determine these values. Response and non-response rates will be analysed using binary logistic regressions, with the PGIC and TGIC scales as dependent variables.
Adverse effect Outcome Analysis: Adverse effects will be quantified through frequency analysis for each item of the NEQ questionnaire, and serious adverse events (SAEs) will be described using descriptive statistics, analysing their correlation with EMDR therapy.
Therapist Influence: The impact of therapists on treatment outcomes will be explored using multivariate models or regression analyses, considering various contextual factors such as different therapy approaches, therapist experience.
Analysis of predictors: Predictors for therapy success will be identified through multivariate regression analyses, using data from multidimensional phenotyping as independent variables.
Feasibility and Practicability Assessment: The practicability of the evaluation process will be assessed by analysing dropout rates, missing values, and the variability of inclusion rates per therapist. An intention-to-treat analysis will be conducted, and satisfaction with the evaluation will be measured using z-scores.

## 5 Discussion

The establishment of the ‘EMDR Pain Network Germany’ is a significant step towards promoting clinical exchange and deepening the understanding of EMDR in pain therapy. This interdisciplinary scientific-clinical platform includes standardised treatment protocols, an online data collection system with anonymised data and suggestions for a comprehensive core assessment set. It also enables regular professionals and promotes collaboration. These components are intended to personalise future therapies and create the basis for large-scale randomised clinical trials to enable systematic research into the mechanisms and effects of EMDR in pain treatment.

The network structure integrates scientists, therapists, and patients and is organised into three main levels: 1. a steering group that leads and organises the network; 2. participating therapists and associated scientists, who keep the network active; and 3. the patient group, which provides outcome data and forms the foundation of the network. This structure serves as a guide and an open system that promotes the continuous expansion of the network and the long-term exchange of experiences and expertise. The integration of these levels introduces considerable complexity, but it is crucial for gaining in-depth and long-term insights into treatment effects and paves the way for the discussion of analytical challenges.

The principle of opening the network to all interested scientists and therapists requires clear inclusion and exclusion criteria. To ensure a balance between the widespread use of EMDR and the maintenance of high-quality standards, basic therapeutic training and an officially recognised training certificate for EMDR (e.g., from the professional association) should be required. This ensures therapeutic standards and the generation of high-quality data for valuable analyses.

The planned analyses, especially predictor analyses, are challenging and require large sample sizes and longer study durations. These requirements fit with current big data initiatives that require early and systematic data collection. A proposed core assessment model will guide future research initiatives and studies, improve comparability and facilitate meta-analytical syntheses. Through continuous data collection and analysis, the network will provide valuable insights into the effectiveness of EMDR in pain management, deepen the understanding of this method and drive future research and clinical trials.

The network not only investigates the effectiveness and prediction of treatment response to EMDR in real clinical settings but also establishes a robust foundation for future randomised controlled multicentre studies. The systematic training and experience of therapists in the use of EMDR, along with standardised data collection methods, facilitate the development of a pool of qualified professionals essential for further clinical research. This pool of therapists will be a valuable resource for future studies aimed at testing the efficacy of EMDR on a larger scale and advancing its implementation in the treatment of complex pain disorders in clinical practice.

The network promotes the use of EMDR in pain therapy without commercial interests. Free working materials and free participation in the network support open science and patient-centred treatment. This approach aims to remove barriers to access and facilitate the dissemination of this effective therapy. This promotes a collaborative and inclusive model that benefits therapists and patients alike.

In summary, the developed framework promises to improve the understanding of the therapeutic effect of EMDR on chronic pain. It enables longitudinal analyses that describe the course of treatment success and identify individual differences in outcomes. This provides the basis for personalised treatment strategies that are tailored to the individual needs of patients with chronic pain in order to optimise therapeutic outcomes and improve quality of life.
