# Supplementary material for: Towards international collaboration of clinical research networks for EMDR: the EMDR Pain Network Germany
Source: Front Psychol. 2024 Oct 4;15:1449150. doi: 10.3389/fpsyg.2024.1449150 (PMC11488483; doi:10.3389/fpsyg.2024.1449150)
Supplement: Supplementary file 1 [file Data_Sheet_1.PDF]

Therapy documentation

Please complete the survey below.

Thank you.

---

1. Number of therapy session ____________________

---

2. Date of session ____________________

---

3. Duration of the therapy session ____________________

---

4. Therapy focus (target)
   - ☐ Stressful Memories
   - ☐ Pain
   - ☐ Fear of Disease
   - ☐ Other

   If other focus, please specify ____________________

---

5. Type of stimulation
   - ☐ Eye Movements
   - ☐ Sound
   - ☐ Tapping
   - ☐ Other

   If other stimulation, please specify ____________________

---

6. SUD before

   ☐ 1 ☐ 2 ☐ 3 ☐ 4 ☐ 5 ☐ 6 ☐ 7 ☐ 8 ☐ 9 ☐ 10

---

7. SUD after

   ☐ 1 ☐ 2 ☐ 3 ☐ 4 ☐ 5 ☐ 6 ☐ 7 ☐ 8 ☐ 9 ☐ 10

---

8. SUP before

   ☐ 1 ☐ 2 ☐ 3 ☐ 4 ☐ 5 ☐ 6 ☐ 7 ☐ 8 ☐ 9 ☐ 10

9. SUP after

☐ 1 ☐ 2 ☐ 3 ☐ 4 ☐ 5 ☐ 6 ☐ 7 ☐ 8 ☐ 9 ☐ 10

---

10. VoC before

☐ 1 ☐ 2 ☐ 3 ☐ 4 ☐ 5 ☐ 6 ☐ 7

---

11. VoC after

☐ 1 ☐ 2 ☐ 3 ☐ 4 ☐ 5 ☐ 6 ☐ 7

---

12. Patient accepts psychological model

Not at all ☐ ☐ ☐ ☐ ☐ Very much

---

13. Patient does homework

Not at all ☐ ☐ ☐ ☐ ☐ Very much

---

14. Patient collaborates during therapy sessions

Not at all ☐ ☐ ☐ ☐ ☐ Very much

---

15. Other remarks ________________________________

---

16. Was this the last session? ☐ Yes

☐ No
