# Supplementary material for: Towards international collaboration of clinical research networks for EMDR: the EMDR Pain Network Germany
Source: Front Psychol. 2024 Oct 4;15:1449150. doi: 10.3389/fpsyg.2024.1449150 (PMC11488483; doi:10.3389/fpsyg.2024.1449150)
Supplement: Supplementary file 2 [file Data_Sheet_2.PDF]

Severe Adverse Events (SAEs)

Notice on the Documentation and Recording of SAEs:

Within the framework of this accompanying study, SAEs particularly include: suicide attempts, self-harming behavior, increased use of drugs (cigarettes, alcohol, other illegal drugs), the necessity of crisis intervention by medical colleagues or family members, hospital stays, or visits to the emergency room. SAEs must be documented regardless of whether they are directly related to the accompanying study or not. All patients are informed about the possible occurrence of SAEs before the start of therapy and are asked to actively report them to their therapist or the study management if they occur. Therefore, active questioning by the therapists is not necessary and should only be done if there is suspicion.

---

1. Were there any SAEs during the treatment period (e.g., suicide attempts, self-harming behavior, increased drug use, crisis intervention, hospitalization)?
   ☐ Yes
   ☐ No

---

2. Where there suicide attempts?
   ☐ Yes
   ☐ No

Do you see a connection to the therapy here?
☐ Yes
☐ No

---

3. Was there self-harming behavior?
   ☐ Yes
   ☐ No

Do you see a connection to the therapy here?
☐ Yes
☐ No

---

4. Was there an increased use of drugs (e.g. cigarettes, alchohol, illegeal drugs)?
   ☐ Yes
   ☐ No

Do you see a connection to the therapy here?
☐ Yes
☐ No

---

5. Where there any crisis interventions by doctores or relatives?
   ☐ Yes
   ☐ No

Do you see a connection to the therapy here?
☐ Yes
☐ No

6. Where there hospitalization or visits to the emergency room?

☐ Yes
☐ No

Do you see a connection to the therapy here?

☐ Yes
☐ No
