# Supplementary material for: Towards international collaboration of clinical research networks for EMDR: the EMDR Pain Network Germany
Source: Front Psychol. 2024 Oct 4;15:1449150. doi: 10.3389/fpsyg.2024.1449150 (PMC11488483; doi:10.3389/fpsyg.2024.1449150)
Supplement: Supplementary file 3 [file Data_Sheet_3.PDF]

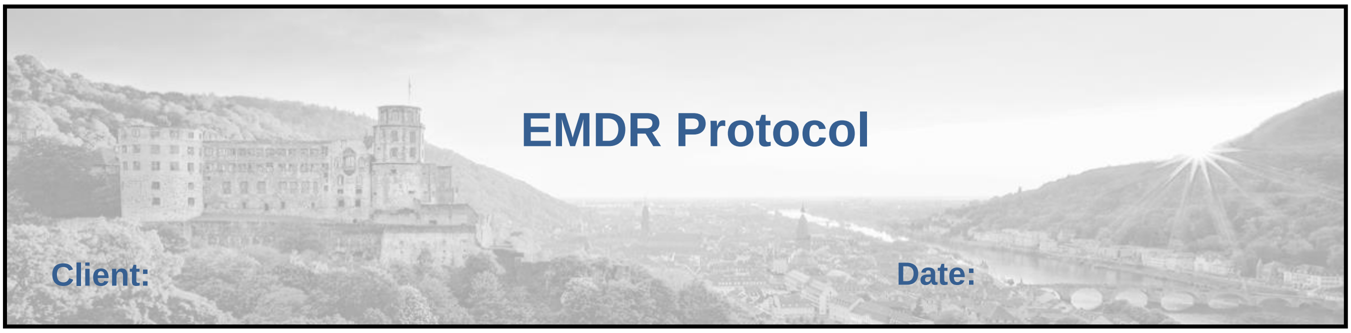

EMDR Protocol

Client: **Date:**

**1.**	**Anamnesis and treatment planning of the session**
**IS (Target)**	**Initial situation (target):**<br>"*Do you agree to work on [one of the 3 possible targets] today?* "<br>a) stressful situation or memory (associated with pain)<br>b) the pain itself<br>c) threat content of pain (pain-associated illness fears/ pain meaning/ illness model).<br><br>____
**2.**	**Stabilization and preparation**
**Building a positive state**	**Building a positive state (source of power) with "key word".**<br>"*Before we proceed to work with the stressful issue, I would like to do some preparatory steps. This includes creating a pleasant state within yourself. At the last appointment, you already learned about various exercises - which one did you like best?* "<br><br>· Positive body resource<br>· Imagination exercise (e.g. light ray exercise, inner place of well-being)<br>· Absorption technique<br><br>Other: ____
**Info**	**Explanation of EMDR:**<br>"*Pain is usually a very effective protective mechanism that has evolved over the course of evolution to protect our bodies and our health. Pain then functions like an alarm system that kicks in when the threat becomes too great. In chronic pain, however, our nervous system has forgotten to distinguish safe from unsafe signals. We then experience pain even though the affected tissue is*

	perfectly healthy. These painful and stressful events then seem to be frozen in the nervous system and in memory.<br>*The eye movements or the other right-left provocations that we work with in EMDR treatment seem to open up access to the nervous system and neural networks and allow the brain to reprocess the experience. This reduces the threat level and allows you to experience the harmless signals from the body as harmless again.*<br>*The important thing to remember throughout all of this work is that your own brain is bringing about the healing and that ultimately you are in control. "*
**Further preparation**	· **Feedback:** "*I now need you to give me brief feedback every now and then on how you are feeling at the moment. I need to know from you exactly what is going on inside you right now. Sometimes things will change, sometimes they won't. I would ask you to describe this to me accurately, without judgment. There are no guidelines and no "right or wrong". Just let whatever happens happen. "*<br><br>· **Stop signal:** "*It is unlikely that the pain or the degree of strain will increase to an unbearable level. But if it does, just raise your hand (therapist shows stop signal with hand) so we can stop the exercise. "*<br><br>· **Chair position** (distance, hand spacing)<br><br>· **At least 2 bilateral provocation modes** (sensory channel, direction, speed).<br>____<br>_
**3.**	**Assessment**
**Image**	**a) Pain-associated stressful memory/situation: Initial image - Intrusive sensory impression.**<br>*"Can you recall the situation again? Which image represents the worst moment of the situation/memory? Where does the situation start and where does it end?"*<br>____<br>____<br>____
	**b) The pain itself: Pain description and pain drawing**<br>***"****Can you recall the pain again? How does it feel?"*<br>If applicable: *"If you focus again completely on the painful area, where exactly does the pain start there, where does it stop, where is it strongest?"*

	▸ Spontaneous pain description:<br><br>▸ Pain description in the categories:<br>Size:<br>Shape:<br>Color:<br>Temperature:<br>Texture:<br>Sound:<br><br>▸ If necessary, let the patient draw the pain (on blank or body sheet)
	**c) Threat content of the pain: initial scene**<br>"*What exactly is the cause of your pain? Which structure is it that hurts? Why does it hurt? What mechanism is the cause? "*<br>Or: *"What meaning does the pain have for you?"*<br><br>▸ Subjective pain model:<br><br>Structure:<br>Defect:<br>Mechanism:<br>Nerve:

	Nervous system:<br><br>**Pain description and pain drawing**<br>▸ Spontaneous pain description:<br><br>▸ Pain description in the categories:<br><br>Size:<br>Shape:<br>Color:<br>Temperature:<br>Texture:<br>Sound:<br><br>▸ If necessary, let the patient draw the pain (on blank or body sheet)
**SUP**	**For b) & c) additionally pain intensity: "***How strong is the pain right now? “*<br><br>"no pain" **0 - 1 - 2 - 3 - 4 - 5 - 6 - 7 - 8 - 9 - 10** "maximum imaginable pain".
**NB**	**Negative self belief (NB):**<br>"*Are there any thoughts about yourself (or in regard to the painful structure) that accompany the pain?"*
**PB**	**Positive self belief (PP):**<br>"*What would you rather say about yourself (or the painful structure) when you think about the pain right now?"*

	___
**VoC**	**VoC (validity of the PB)**<br>"*How valid or true does [PB] feel?*"<br><br>"completely false" **1 - 2 - 3 - 4 - 5 - 6 - 7** "completely true"
**Emotion**	**Emotions:**<br>"*When you imagine the image/pain along with the negative thought about yourself [repeat NB], what feelings arise in you now?* "<br><br>___
**SUD**	**Subjective units of distress (SUD)**<br>"*How burdened are you by these emotions right now?* "<br><br>"*no distress*" **0 - 1 - 2 - 3 - 4 - 5 - 6 - 7 - 8 - 9 - 10** "*maximum distress*"
**BF**	**Localizing the body feelings (BF):**<br>*"Where do you feel it in your body?"*<br><br>___<br>**For b) & c) pain location, "***Where do you feel the pain in your body?* "<br><br>___
**4.**	**Working through**
**Image + NB+ Emotion + BF**	**Entry with picture, NB, emotion and BF**<br>*"Please imagine the image [repeat image] or the pain [repeat pain description] along with the negative thought [repeat NB] and pay attention to how this feels [repeat emotion] and where you feel it in your body [repeat BF].*<br>*Are you in contact with it? If so, please nod briefly.*<br>*Stay with it and please follow my finger with your eyes."*
**BPS**	**Start of the bilateral provocation series**
**Break + feedback**<br><br>**Record:**<br><br>**-Associations**	**After each set:**<br>**Pause:** "*Take a deep breath"*<br>**Feedback:** *"What do you perceive?".... "Go on with that."*<br><br>___<br>___<br>___

**- Number of Set**<br><br>**- E = Eye-movements/ T = Tapping**	

	____<br>____<br>____<br>____
	**In case of blockages/ gyrations (2 sets without changes):**<br><br>**Stimulate, push process:**<br>- Extend set, change direction of eye movements, change type of provocation<br>- Focusing (revisiting the initial topic) or changing the focus<br>- Affect bridge (going back to background experiences)<br>- Change level of perception ("*If your body could talk, ...", "Where do you feel this in your body right now?* “)<br><br>**Decelerate:**<br>- Body resource ("*I ask you to put your hand on the spot [...]*"), imaginative resource<br>- Parts work, symbolizing content ("*When the pain [...]", "When you were a child [...]*")<br>- Distancing, change of perspective ("*If you look at the scene today from a distance/ as an adult person [...]*")<br>- Support (Encourage, Allow, Recognize)<br><br>**In case of flooding/dissociation:**<br>- Strengthen therapeutic dyad<br>- Control, build security<br>- Returning to reality (grounding) by addressing, touching - establishing a relationship to the here and now
	**At the end of an association channel** (2x positive or neutral feedback):<br>Ask client to go back to the initial situation/pain.<br>"*What are you noticing now?* “<br><br>____
**SUP**	**For b) & c) additionally pain intensity: "***How strong is the pain right now?* “<br><br>"no pain" **0 - 1 - 2 - 3 - 4 - 5 - 6 - 7 - 8 - 9 - 10** "maximum imaginable pain".
	Whatever the client responds: a set of bilateral stimuli: "*Stay with it, follow the eye movements!* “<br><br>If more distressing material comes, continue to process it:<br>"*What else do you need to make it a 0?* “

**Further BPS**	
**5.**	**Anchoring**
**Re-evaluation (SUD)**	**When nothing more distressing comes up/ you need an intermediate orientation/ the session is coming to an end:**<br>· Ask client to go back to the initial situation<br>· Current level of distress (SUD) with regard to initial situation/pain:<br><br>*"no distress"* **0 - 1 - 2 - 3 - 4 - 5 - 6 - 7 - 8 - 9 - 10** *"maximum distress"*
**SUD = 0**	**End complete session**<br>· Review PB: "*Is the positive belief [repeat PB] still true or is there another one now that fits even better?* "<br><br>· **VoC (validity of PB):**<br>*"completely false"* **1 - 2 - 3 - 4 - 5 - 6 - 7** *"completely true"*<br><br>· If VoC < 6 : "*What is missing for it to be a 7*"?<br>Process until VOC = 7; then make a set of eye movements with initial situation *[repeat]* and PB *[repeat].*<br><br>· Anchoring the PB with the initial situation by means of 2-3 sets of slow bilateral provocations.<br>"*Think again about the distressing memory/pain and at the same time think about this positive thought.* "

**SUD > 0**	**End incomplete session**<br>• Obtain and give feedback on the therapy session<br><br>E.g.: "*We have reached the end of the hour. How do you feel about the idea of slowly ending the session? Is there anything you took away or learned for yourself today?* "<br>• Does the client need anything else?<br><br>• Find positive closure<br><br>▪ e.g.: by suggesting exercises (vault exercise followed by feel-good place exercise)<br>▪ or: "*Direct your attention inward. Feel into your body and perceive what is there without judging it. Which part of your body feels good and safe to you? Now focus completely on this one pleasant sensation in your body and the thought [repeat PB or introduce thought that the body is perfectly healthy, resilient and intact]. Trust your body and the sensations from your body. Please feel carefully inside, notice this pleasant feeling of well-being in your body and at the same time follow my finger movements [2-3 slow bilateral stimulation sets] - let happen whatever happens."*
	**Body test** (optional)
**Body Scan**	**If SUD=0 and VoC=7** (not applicable if there is no/slight improvement or if there is no more time).<br>*"Please close your eyes, imagine the initial situation, tell yourself the PB [repeat], and go through your whole body internally. Tell me if you feel anything else while doing this."*<br><br>· If negative body sensations are reported: Work through until the discomfort disappears or try resource activation (e.g., light ray method, antidote exercise).<br><br>· If a positive and a negative body feeling are reported: *"Stay in touch with the positive feeling and just let the other one resonate. "*
**7.**	**Conclusion**
**Info**	**Information on the process between sessions**<br>· *"We are now at the end of today's therapy session....* "<br>· Continuation of working through in the form of conscious and unconscious processes (insights, thoughts, memories, dreams)<br>· Favorable handling: observe, record in diary, bring to next session, brief feedback to therapist (e.g. email).
**Balance**	**Establish a balanced state** (distancing and/or resource activation)

	· Imagination exercise (vault exercise, feel-good place, light ray technique).<br>· Positive body resource<br>· Absorption technique<br>· Other:<br>______________________________
**8.**	**Next session**
**Feedback on last session**	**Ask patient for feedback on therapy**<br>*"Are you satisfied with our treatment?", "How are you doing in your daily life?", "Do you recognize any development?"*<br>______________________________
**Initial image of the last session**	**Call current image for the initial situation of the last session**<br>*"What happened to the image/pain from last time?"*<br>______________________________
**SUD**	**Subjective level of distress (SUD)**<br>*"no distress"* **0 - 1 - 2 - 3 - 4 - 5 - 6 - 7 - 8 - 9 - 10** *"maximum distress"*
**VoC**	**VoC (validity of the PB)**<br>*"completely false"* **1 - 2 - 3 - 4 - 5 - 6 - 7** *"completely true"*
**Notes:**	

NB: negative belief, PB: positive belief, VoC: Validity-of-Cognition Scale, SUD: Subjective-Units-of-Distress Scale, BPS: Bilateral Provocation Series.
